# Individual and joint association of adulthood experiences and parental or teacher smoking with adolescent cigarette smoking

**DOI:** 10.18332/tid/127519

**Published:** 2020-10-01

**Authors:** Heewon Kang, Sung-il Cho

**Affiliations:** 1 Institute of Health and Environment, Seoul National University, Seoul, Republic of Korea; 2 Department of Public Health Sciences, Graduate School of Public Health, Seoul National University, Seoul, Republic of Korea

**Keywords:** cigarette smoking, adolescents, tobacco use, adulthood experience, significant adults

## Abstract

**INTRODUCTION:**

One limitation of the minimum legal age (MLA) law, the primary strategy for curbing youth smoking, is the rite-of-passage effect. Adulthood experiences and the behavior of surrounding adults help build ‘adult identity’ among adolescents. We examined the individual association of adulthood experience and joint association with significant adults who smoke with adolescent cigarette smoking.

**METHODS:**

A nationally representative cross-sectional sample of 138542 South Korean adolescents aged 12–18 years (mean: 15 years) from the 2014 and 2015 Korea Youth Risk Behavior Survey was used. Adulthood markers used were tall stature, precocious sexual development, independent living, and job experience. Parents and teachers were considered significant adults. Logistic regression analyses and relative risk due to interaction (RERI) calculations were conducted.

**RESULTS:**

Tall stature (OR=1.19; 95% CI: 1.08–1.31), precocious sexual development (OR=1.51; 95% CI: 1.36–1.69), independent living (OR=1.24; 95% CI: 1.08– 1.43), and job experience (OR=4.38; 95% CI: 4.14–4.64) were associated with cigarette smoking among study participants. Statistically significant additive interactions were found for parental smoking and job experience (RERI=0.41; 95% CI: 0.06–0.76), teacher smoking and precocious development (RERI=0.71; 95% CI: 0.28–1.15), teacher smoking and independent living (RERI=0.68; 95% CI: 0.11–1.24), teacher smoking, and job experience (RERI=2.12; 95% CI: 1.66– 2.58).

**CONCLUSIONS:**

The association between adulthood experience and adolescent cigarette smoking suggests the rite-of-passage effect, which may be strengthened by the MLA law. Raising the MLA to an age much higher than the normative age of adulthood initiation is required. Additionally, targeted intervention for adolescents with both adulthood experience and exposure close to adult smoking are required to curb youth smoking.

## INTRODUCTION

The purpose of limiting the age for purchasing cigarettes is to curb youth smoking initiation. Yet, a number of drawbacks to the minimum legal age (MLA) law on tobacco sales and supplies have been identified^1^. One of the greatest dangers of the age limit on cigarette sales is that adolescents may use cigarettes to appear grownup or older than their chronological age^2^. The correspondence between the MLA to purchase tobacco products, typically set at 18–22 years^3^, and the normative age of adulthood initiation, may inadvertently lead adolescents to think cigarette smoking is a milestone of maturity. In this way, smoking may serve as a rite-of-passage into adulthood^4^. Rite-of-passage is a landmark event of transitioning from one group to enter another. Adolescents often attempt to project the appearance of approaching adulthood. This aspiration is associated with a variety of behaviors, including smoking^4^. A qualitative study of Korean male adolescents identified ‘imitating adults with curiosity’ as a reason for smoking experimentation^5^. Adolescents from other cultural backgrounds, such as China, also reported smoking enhances one’s maturity^6^. Although decreased to a noticeable extent from 28% in 2001 to 17% in 2015, Californian adolescents’ perception of smoking as a milestone for maturity is still prevalent^7^.

Countries that have ratified the Framework Convention on Tobacco Control are obliged to restrict youth access to tobacco products. In Korea, the prohibition of tobacco sales to minors, introduced in 1995, is considered one of the key policies particularly targeting adolescents, along with the universal coverage of school-based smoking prevention and cessation programs in 2015^8^. However, the efficacy of the MLA is debated. In 2018, purchasing from stores was the most common means of acquiring tobacco products, and more than 70% of Korean adolescents could purchase cigarettes without difficulty^9^. Consequently, approximately 7% of Korean adolescents are current users of cigarettes^9^. Kang et al.^10^ suggested strengthening enforcement with measures such as age verification and penalties for non-compliance.

Nonetheless, beyond the enforcement of the instrumental measure alone, the efficacy of any law relies on the interactions among personal and social influences^11^. Grucza et al.^12^ examined the instrumental efficacy of the MLA law using quasi-experimental methods, but did not examine the individual and social influences related to the law. As mentioned, setting the MLA as the age of adulthood initiation may provide an erroneous perception that smoking is a symbol of maturity. Among the personal influences that may affect the efficacy of the MLA law are experiences of adulthood^13^, as these experiences allow adolescents to feel as if they have reached adulthood^14^. A previous study has identified a number of categories of adulthood markers^15^. First, age-related or biological experiences, which include being able to bear children and reaching full height. Second, social experiences associated with role transitions, including independent residence and being employed. Finally, family capacities such as marriage and parenting have been identified as adulthood experiences.

Social forces that affect the efficacy of the MLA law derive from the influence of surrounding adults. Adolescents often incorporate various characteristics of close adults, such as parents and teachers, into their own adult identities^16^. Adolescents are most often exposed to the thoughts and behaviors of their parents. Teachers are also considered critical as they guide adolescents in schools, a key developmental arena. These significant adults act as role models to adolescents by showing certain behaviors that are expected and appropriate in adulthood. Therefore, significant adults’ use of cigarettes may have a normative effect on how adolescents perceive smoking in adulthood. Thus, adolescents who go through some adulthood experiences and are exposed to adults who smoke would easily assume they have begun their entry into adulthood and should incorporate adult behaviors (i.e. smoking). If this is true, adolescents with adulthood experiences plus exposure to significant adults who smoke would be at greater risk of smoking than would those with separate risks of adulthood experiences or exposure to significant adults who smoke. Put differently, there would be an additive interaction effect of adulthood experiences and significant adults’ smoking on the risks of adolescent smoking.

Adulthood experiences during adolescence have been found to be positively associated with adolescent smoking in various studies. However, these studies have generally focused on a single marker of adulthood such as job experience^17^ or precocious sexual development^18^. Only in young adults has been conducted a simultaneous assessment of the effects of multiple adulthood experiences on smoking^19^. Moreover, to the best of our knowledge, no study has examined the interactions among various adulthood experiences during adolescence and smoking by significant adults, and their effects on adolescent cigarette smoking. Specifically, examining additive interactions can be useful. Compared to multiplicative interactions, additive interactions are a more relevant measure for public health as they help identify subgroups that would benefit most from targeted interventions^20^. Because experiences of adulthood during adolescence are not uncommon, unexpected, or abnormal, and can therefore not be directly controlled, this study aimed to identify alternative solutions.

This study was designed to investigate the personal and social influences on smoking that impact the efficacy of the MLA law. Using a nationally representative sample of South Korean adolescents, this study aimed to: 1) investigate the association of adulthood experiences with adolescent cigarette smoking, and 2) assess the additive interactive effect of adulthood experiences and parents or teachers who smoke on adolescent cigarette smoking.

## METHODS

### Data source and study participants

A total of 140103 South Korean adolescents aged 12–18 years (grades 7–12) completed the 10th (2014) and 11th (2015) Korea Youth Risk Behavior Survey (KYRBS). The 2014 and 2015 surveys were pooled based on the availability of variables necessary for this study. KYRBS is a nationally representative online survey with a participation rate of 97.2% in 2014 and 96.7% in 2015. Further details on the data, study design, outcome measures, and participants, have been described elsewhere^21^. One of the study aims was to examine the impact of parental smoking, so adolescents with no parents were excluded from the analysis based on the study eligibility criteria (n=1561). Finally, 138542 adolescents were included as study participants. This study was exempt from review by the Seoul National University Institutional Review Board.

### Measures

Participants were asked: ‘Have you ever smoked a cigarette, even one or two puffs?’. Those who responded ‘yes’ were then asked: ‘During the past 30 days, on how many days did you smoke cigarettes, even one cigarette?’. Participants who responded ‘1–2 days,’ ‘3–5 days,’ ‘6–9 days,’ ‘10–19 days,’ ‘20–29 days,’ or ‘every day’ were classified as current smokers.

The markers of adulthood described by Arnett^15^ were used. However, family capacities related to marriage and parenthood were excluded, as the study participants were all school-attending adolescents and were unlikely to be married or have children. The adulthood experiences examined were tall stature, precocious sexual development, living away from parents, and having job experience. Tall stature was defined as height above the 95th percentile for age and sex. Precocious sexual development was defined as being 10 years old or younger when first experiencing menarche (girls) or spermarche (boys), corresponding to the 5th percentile for the age of menarche and spermarche. Participants were asked whether they lived with a parent to identify those living away from parents. Participants who reported living with their mother, father, or both, were defined as living with parents; those who reported not living with either parent were defined as living away from parents. Job experience was defined as having a parttime job in the past 12 months.

In this study, parents and teachers were considered significant adults. Parental smoking was defined as having a father and/or mother who smoked. Teacher smoking was defined as having observed teachers or school staff smoking outside the school building in the past 30 days.

Covariates used in this study were age (continuous variable, 12–18 years), sex (boys, girls), school type (middle school, general high school, vocational high school), perceived household wealth (high, middle, low), perceived academic performance (high, middle, low), close friends smoking (yes, no), and having received in-school smoking prevention education in the past 12 months (yes, no).

### Statistical analyses

The characteristics of the study participants are presented as unweighted frequencies and weighted percentages according to current cigarette smoking status, as all analyses conducted considered the complex sampling design of the survey. Age is presented as mean ± standard deviation.

Multiple logistic regression models were used to estimate the odds ratios (OR) and 95% confidence intervals (CIs) for the association between current cigarette smoking and adulthood experiences. In Model 1, the impact of adulthood experiences was examined with adjustment of the *a priori* confounders described above. Models 2 and 3 were further adjusted for parental smoking and teacher smoking, respectively.

To evaluate the additive interaction between individual adulthood experiences and smoking by significant adults, four subgroups were created for each adulthood experience and significant adult smoker. For example, the subgroups for part-time job and parental smoking were: 1) participants with no job experience and non-smoking parents (reference group), 2) participants with no job experience and smoking parents (OR_A_), 3) participants with job experience and non-smoking parents (OR_B_), 4) participants with job experience and smoking parents (OR_AB_). The additive interaction of adulthood experiences and significant adult smoking was estimated using the relative excess risk due to interaction (RERI), which can be expressed^20^ as OR_AB_ – OR_A_ – OR_B_ + 1. RERI >0 with a 95% CI lower limit >0 indicates the effects arising from the additive interaction of adulthood experience and significant adult smoking has a greater effect than the sum of adulthood experience and significant adult smoking alone. CIs for the RERI measure were calculated using the delta method. Missing values for the variables used in our study ranged from 0.3% for school type to 2.7% for height. To preserve the sampling structure and accurate parameter estimation, missing data were treated as not missing completely at random by specifying the NOMCAR option in SAS.

### Sensitivity analyses

To identify whether the impact of adulthood experience and parental or teacher smoking on adolescent smoking differed by the frequency of smoking, we additionally assessed whether adulthood experience and close adult smoking were associated with daily smoking among adolescent participants. Daily smokers were defined as those who responded ‘every day’ to the question: ‘During the past 30 days, on how many days did you smoke cigarettes, even one cigarette?’. All other measures were identical to the main analyses.

## RESULTS

### Characteristics of participants by current smoking status

The characteristics of participants according to their cigarette smoking status are summarized in Table 1. Of all participants, 91.7% were not current smokers, and 8.3% were current smokers. Participants with tall stature (≥95th percentile) comprised 5.5% non-smokers and 6.5% smokers. Precocious sexual development characterized 4.0% of non-smokers and 6.9% of smokers. Of non-smokers, 1.6% lived away from their parents, and 3.2% of current smokers did so. Among non-smoking adolescents, 10.0% had job experience compared with 48.6% of current smokers. Among non-smokers, 45.6% had at least one smoking parent, whereas 53.2% of current smokers did. Finally, 35.9% of non-smokers and 59.8% of current smokers had observed teachers or school staff smoking.

**Table 1:** Characteristics of the study participants by smoking status, data from 2014 and 2015 Korea Youth Risk Behavior Survey (N=138542)

*Characteristics*	*Total*	*Non-current smokers*	*Current smokers*
	*n (%)*	*n (%)*	*n (%)*
**Total**	138542 (100.0)	127544 (91.7)	10998 (8.3)
**Age** (years), mean ± SD	15.0 ± 0.01	15.0 ± 0.02	16.0 ± 0.02
**Sex** (female)	67901 (48.0)	65564 (50.5)	2337 (19.9)
**School type**			
Middle	69623 (47.7)	67061 (50.0)	2562 (21.9)
General high	56412 (43.1)	50729 (42.3)	5683 (52.8)
Vocational high	12104 (9.2)	9407 (7.7)	2697 (25.3)
**Household wealth**			
High	47971 (35.0)	44768 (35.5)	3203 (29.6)
Middle	66515 (47.7)	61640 (48.1)	4875 (43.8)
Low	24056 (17.3)	21136 (16.5)	2920 (26.6)
**Academic performances**			
High	51891 (37.4)	49436 (38.7)	2455 (22.4)
Middle	38845 (28.1)	38322 (28.5)	2523 (23.2)
Low	47806 (34.6)	41786 (32.8)	6020 (54.4)
**Close friends smoking**	59089 (44.3)	48619 (39.7)	10470 (95.4)
**Smoking prevention education**	82426 (58.7)	75846 (58.7)	6580 (58.9)
**Tall stature** (≥95th percentile)	7455 (5.6)	6788 (5.5)	667 (6.5)
**Precocious sexual development**	5852 (4.2)	5099 (4.0)	753 (6.9)
**Living away from parents**	2725 (1.8)	2353 (1.6)	372 (3.2)
**Having a job experience**	17945 (13.2)	12596 (10.0)	5349 (48.6)
**Parental smoking**	64926 (46.2)	59031 (45.6)	5895 (53.2)
**Teacher smoking**	52854 (37.9)	46308 (35.9)	6546 (59.8)

*Frequency missing: school type (n=403), age (n=526), height (n=3687). Variables with missing data were treated with the NOMCAR option.

### Impact of adulthood experience and smoking by significant adults on adolescent smoking

Several associations between adulthood experiences and current cigarette smoking were identified (Table 2). In Model 1, tall stature (OR=1.19; 95% CI: 1.08– 1.31), precocious sexual development (OR=1.51; 95% CI: 1.36–1.69), living away from parents (OR=1.24; 95% CI: 1.08–1.43), and having job experience (OR=4.38; 95% CI: 4.14–4.64) were associated with smoking. Adjustment for parental or teacher smoking did not essentially change the estimated effects of adulthood experiences (Models 2 and 3).

**Table 2:** Association of adulthood experiences and significant adults smoking with current cigarette smoking status, data from 2014 and 2015 Korea Youth Risk Behavior Survey (N=138542)

	*Current cigarette smoking*		
	*Model 1^a^*	*Model 2^b^*	*Model 3^c^*
	*AOR (95% CI)*	*AOR (95% CI)*	*AOR (95% CI)*
**Adulthood experiences**			
Tall stature	1.19 (1.08–1.31)	1.19 (1.08–1.31)	1.16 (1.06–1.28)
Precocious sexual development	1.51 (1.36–1.69)	1.52 (1.36–1.69)	1.48 (1.32–1.65)
Living away from parents	1.24 (1.08–1.43)	1.24 (1.08–1.43)	1.24 (1.07–1.43)
Job experience	4.38 (4.14–4.64)	4.36 (4.12–4.61)	4.29 (4.05–4.54)
**Significant adult smoking**			
Parental smoking		1.16 (1.11–1.22)	
Teacher smoking			1.59 (1.51–1.68)

a Model 1: adjusted for age (continuous), sex (male/female), school type (middle/general high/vocational high), close friends smoking (yes/no), participation in smoking prevention education (yes/no), perceived household wealth (high/mid/low), and perceived academic performance (high/mid/low).

b Model 2: additionally adjusted for parental smoking (yes/no) to Model 1.

c Model 3: additionally adjusted for teacher smoking (yes/no) to Model 1. AOR: adjusted odds ratio.

### Additive interaction between parental smoking and adulthood experience

Estimates of the interactive effects of individual adulthood experiences and parental smoking on adolescent cigarette smoking are presented in Table 3. Compared to non-smoking parents and shorter stature, there was an increased OR for current smoking among adolescents with smoking parents and shorter stature (OR=1.22; 95% CI: 1.16–1.28), non-smoking parents and tall stature (OR=1.38; 95% CI: 1.21– 1.58), and smoking parents along with tall stature (OR=1.37; 95% CI: 1.21–1.56). Compared to nonsmoking parents and normal development, the ORs for other subgroups were 1.21 (95% CI: 1.16–1.27) for smoking parents and normal sexual development, 1.68 (95% CI: 1.45–1.95) for non-smoking parents and precocious sexual development, and 1.83 (95% CI: 1.58–2.13) for smoking parents and precocious development. Compared to participants who lived with non-smoking parents, the ORs were 1.21 (95% CI: 1.16–1.27) for smoking parents and living with parents, 1.51 (95% CI: 1.23–1.86) for non-smoking parents and living away from parents, and 1.47 (95% CI: 1.21–1.79) for smoking parents and living away from parents. Compared to participants with nonsmoking parents and no job experience, the ORs for each group were 1.18 (95% CI: 1.12–1.25) for smoking parents and no job experience, 4.49 (95% CI: 4.18–4.82) for non-smoking parents and job experience, and 5.08 (95% CI: 4.72–5.47) for smoking parents and job experience.

**Table 3:** Additive interactive effect of adulthood experiences and parental smoking on the risk of adolescent current cigarette smoking, data from 2014 and 2015 Korea Youth Risk Behavior Survey (N=138542)

*Adulthood experiences*	*Current cigarette smoking*				*RERI (95% CI), p*
	*Non-smoking parents*		*Smoking parents*		
	*Non-current/ current*	*AOR ^a^ (95% CI)*	*Non-current/ current*	*AOR ^a^ (95% CI)*	
**Tall stature^a^**					
No	63344/4448	1 (Ref.)	54281/5327	1.22 (1.16–1.28)	-0.23 (-0.48–0.02), 0.073
Yes	3625/339	1.38 (1.21–1.58)	3163/328	1.37 (1.21–1.56)	
**Sexual development^b^**					
Normal	65891/4705	1 (Ref.)	56554/5540	1.21 (1.16–1.27)	-0.06 (-0.42–0.31), 0.761
Precocious	2622/398	1.68 (1.45–1.95)	2477/355	1.83 (1.58–2.13)	
**Residential status^c^**					
With parents	67426/4929	1 (Ref.)	57765/5697	1.21 (1.16–1.27)	-0.25 (-0.66–0.17), 0.251
Away from parents	1087/174	1.51 (1.23–1.86)	1266/198	1.47 (1.21–1.79)	
**Job experience^d^**					
No	62626/2743	1 (Ref.)	52322/2906	1.18 (1.12–1.25)	0.41 (0.06–0.76), 0.022
Yes	5887/2360	4.49 (4.18–4.82)	6709/2989	5.08 (4.72–5.47)	

Covariates adjusted for all models are: age (continuous), sex (male/female), school type (middle/general high/vocational high), close friends smoking (yes/no), participation in smoking prevention education (yes/no), perceived household wealth (high/mid/low), and perceived academic performance (high/mid/low).

a Adjusted for sexual development (normal/precocious), residential status (with/away from parents), job experience (yes/no) and the covariates.

b Adjusted for tall stature (yes/no), residential status (with/away from parents), job experience (yes/no) and the covariates.

c Adjusted for tall stature (yes/no), sexual development (normal/precocious), job experience (yes/no) and the covariates.

d Adjusted for tall stature (yes/no), sexual development (normal/precocious), residential status (with/away from parents) and the covariates. AOR: adjusted odds ratio. RERI: relative excess risk due to interaction.

Job experience and parental smoking had an additive interactive effect on current cigarette smoking, with a RERI of 0.41 (95% CI: 0.06–0.76). This result suggests there would be 0.41 excess relative risk due to the additive interaction effect of job experience and parental smoking on adolescent smoking. No other statistically significant additive interactions were identified.

### Additive interaction between teacher smoking and adulthood experience

Estimates for the joint association of individual adulthood experiences and teacher smoking on adolescent cigarette smoking are presented in Table 4. Compared with participants who were not exposed to teacher smoking and were not tall, the ORs for current smoking in each group were 1.68 (95% CI: 1.60–1.78) among those who were exposed to teacher smoking and were shorter, 1.23 (95% CI: 1.06–1.42) among those unexposed to teacher smoking who were tall, and 2.04 (95% CI: 1.80–2.31) among those exposed to teacher smoking who were tall. Compared to those who were not exposed to teacher smoking and had normal sexual development, the ORs for cigarette smoking were 1.66 (95% CI: 1.58–1.76) for those exposed to teacher smoking and exhibiting precocious development, 1.40 (95% CI: 1.18–1.66) for those unexposed to teacher smoking and having precocious development, and 2.78 (95% CI: 2.42– 3.19) for those exposed to teacher smoking and experiencing precocious development. Compared with non-exposure to teacher smoking and living with parents, the ORs were 1.68 (95% CI: 1.59– 1.77) for those exposed to teacher smoking and living with parents, and 2.50 (95% CI: 2.05–3.06) for those exposed to teacher smoking and living away from parents. Compared to non-exposure to teacher smoking and no job experience, the ORs were 1.58 (95% CI: 1.49–1.68) for those exposed to teacher smoking and no job experience, 4.23 (95% CI: 3.89– 4.60) for those unexposed to teacher smoking and having job experience, and 6.93 (95% CI: 6.42–7.47) for those exposed to teacher smoking and having job experience.

**Table 4:** Additive interactive effects of adulthood experiences and teacher smoking on the risk of adolescent current cigarette smoking, data from 2014 and 2015 Korea Youth Risk Behavior Survey (N=138542)

*Adulthood experiences*	*Current cigarette smoking*				*RERI (95% CI), p*
	*Has not seen teacher smoking*		*Seen teacher smoking*		
	*Non-current/ current*	*AOR ^a^ (95% CI)*	*Non-current/ current*	*AOR ^a^ (95% CI)*	
**Tall stature^a^**					
No	75155/4038	1 (Ref.)	42470/5737	1.68 (1.60–1.78)	0.13 (-0.18–0.43), 0.415
Yes	4074/243	1.23 (1.06–1.42)	2714/424	2.04 (1.80–2.31)	
**Sexual development^b^**					
Normal	78120/4225	1 (Ref.)	44325/6020	1.66 (1.58–1.76)	0.71 (0.28–1.15), 0.001
Precocious	3116/227	1.40 (1.18–1.66)	1983/526	2.78 (2.42–3.19)	
**Residential status^c^**					
With parents	79761/4323	1 (Ref.)	45430/6303	1.68 (1.59–1.77)	0.68 (0.11–1.24), 0.019
Away from parents	1475/129	1.15 (0.92–1.45)	878/243	2.50 (2.05–3.06)	
**Job experience^d^**					
No	74352/2453	1 (Ref.)	40596/3196	1.58 (1.49–1.68)	2.12 (1.66–2.58), <0.001
Yes	6884/1999	4.23 (3.89–4.60)	5712/3350	6.93 (6.42–7.47)	

Covariates adjusted for all models are: age (continuous), sex (male/female), school type (middle/general high/vocational high), close friends smoking (yes/no), participation in smoking prevention education (yes/no), perceived household wealth (high/mid/low), and perceived academic performance (high/mid/low).

a Adjusted for sexual development (normal/precocious), residential status (with/away from parents), job experience (yes/no) and the covariates.

b Adjusted for tall stature (yes/no), residential status (with/away from parents), job experience (yes/no) and the covariates.

c Adjusted for tall stature (yes/no), sexual development (normal/precocious), job experience (yes/no) and the covariates.

d Adjusted for tall stature (yes/no), sexual development (normal/precocious), residential status (with/away from parents) and the covariates.

AOR: adjusted odds ratio. RERI: relative excess risk due to interaction.

Additive interactive effects were observed for exposure to teacher smoking with sexual development (RERI=0.71; 95% CI: 0.28–1.15), residential status (RERI=0.68; 95% CI: 0.11–1.24), and job experience (RERI=2.12; 95% CI: 1.66–2.58). These results indicate excess relative risks of 0.71, 0.68, and 2.12 due to the additive interactions between teacher smoking and sexual development, residential status, and job experience, respectively.

### Sensitivity analyses

The results of sensitivity analyses are shown in Tables S1–S4 in the Supplementary File. The adulthood experiences considered in this study were all positively associated with adolescent daily cigarette smoking. As in the main analyses, we found an additive interaction between parental smoking and part-time jobs. We also found additive interactions between teacher smoking and living away from parents and having job experience. However, the additive interaction between teacher smoking and precocious sexual development was lost in sensitivity analyses.

## DISCUSSION

Using a nationally representative sample of South Korean adolescents, the independent effects of adulthood experiences and the interactive effects of adulthood experiences with smoking by significant adults on adolescent cigarette smoking were evaluated. Adolescents with adulthood experiences showed increased risks of cigarette smoking, and those with job experiences showed a particularly elevated risk. Additive interaction effects were identified for adolescents who were exposed to teacher smoking, precocious sexual development, living away from parents, and job experience.

This study builds on the existing literature describing multiple biological and social markers of adulthood^15^. The finding that adulthood experiences such as precocious sexual development^18^, living away from parents^22^ and having part-time job experience^17^ were associated with smoking is supported by evidence from previous longitudinal studies. Previous research considering various precocious transition events also showed early full-time work, sexual activity, leaving home, and cohabitation increased the risk of smoking^19^.

The association between precocious sexual development and cigarette smoking may be biologically based. Evidence suggests the development of the socioemotional network, which is crucial for reward processing, occurs around the time of puberty, heightening the adolescents’ risk-taking behaviors^23^. In addition, the cognitive control network, which controls impulsive behaviors, develops gradually from adolescence until early adulthood^23^. Psychological evidence suggests adolescents maturing earlier than their peers have lower self-esteem and higher stress levels^24^. Interpersonal factors also matter, as these early maturing adolescents may adopt behaviors such as smoking to bond with those of similar developmental age (i.e. older adolescents or adults)^24^.

The increased risk of substance use among adolescents living outside their parental homes may be attributed to decreased parental monitoring, supervision, and quality time spent with family^19^. Most previous studies examining the impact of living arrangements on adolescent cigarette smoking have focused on family intactness (i.e. living with both parents vs one parent)^25^. However, this study investigated the impact of living apart from both parents as a marker of adulthood and did not differentiate between living with one parent or both.

Among the adulthood experiences investigated in this study, job experience had the greatest effect on current smoking. Although there may be some benefit to working during adolescence, including developing confidence and work-related skills, considerable evidence indicates an association between working and substance use during adolescence^26^. The precocious development perspective suggests employment during adolescence, which is similar to an adult worker role, leads adolescents to adopt adult-like behaviors^17^. Increased sensory cues and exposure to secondhand smoke (SHS) from smoking adults at work can also contribute to the increased risk of adolescent smoking^27^. Furthermore, according to the time tradeoff perspective, adolescents who are employed have fewer opportunities to engage in experiences critical for development, such as schoolwork, extra-curricular activities, and hobbies, and are more likely to engage in impulsive and risky activity, such as smoking^17^. Earned income can also increase access to tobacco products among adolescents^27^. This result points to the importance of workplace intervention to curb youth smoking. Smoking among working adolescents was associated with exposure to SHS at work^28^. Thus, comprehensive control programs, particularly targeting both adolescents and their adult colleagues in places where adolescents work, may be required.

Previous studies have assessed whether cigarette smoking limits adolescents’ growth, with mixed results showing no association^29^ and decreased height^30^. The positive relationship between height and cigarette smoking identified in this study is not aligned with the results of previous longitudinal studies. Given the cross-sectional design of this study, these findings may be explained by reverse causation. However, as stature higher than the 95th percentile of the same age and sex is unlikely to be merely caused by smoking, it is possible that the exceptionally tall stature influenced smoking behaviors in adolescents.

The rite-of-passage impact of smoking can be inferred from the positive association of adulthood markers and cigarette smoking. Importantly, the study results highlight the role of significant adults in these associations. Job experience was one adulthood marker that showed an additive interaction with both parental and teacher smoking. Having a job is a symbolic marker of the transition from adolescence to adulthood, which can lead adolescents toward adult roles and responsibilities^31^. More than any of the other adulthood experiences considered in this study, job experience exposed adolescents to more adult-centered environments, which may have led adolescents to view themselves as adults^31^. These findings suggest working adolescents who perceive themselves as adults are more likely to adopt the smoking behaviors of significant adults.

This study identified several interactions between adulthood experiences and exposure to teachers’ smoking. Precocious sexual development, living away from parents, and having a job showed additive interactions with teacher smoking. A study on adolescents in Denmark concluded that teachers’ smoking had a greater impact on heavy smoking in adolescents than did that of parents^32^. School plays a critical role in adolescent development, including shaping their health behaviors. Therefore, knowing or observing teachers who smoke in schools can reduce adolescents’ negative attitudes toward smoking^33^ and may increase smoking among adolescents. These results emphasize the need for smoking prevention and cessation programs targeting school personnel, as well as the implementation and strict enforcement of school policies on tobacco use. School staff members should quit smoking cigarettes or at least smoke in places where they cannot be seen. These measures will not only protect the health of teachers and staff but also that of adolescents through appropriate role modeling.

Increasing the MLA is a proven effective measure for preventing smoking^34^. However, the present results indirectly demonstrate potential drawbacks of the MLA. We have not measured the effects of the MLA directly. Yet, as it is important to consider the personal and social influences, when evaluating the efficacy of laws^11^, our results carefully suggest there may be a loophole in the law. Such a loophole may be avoided by raising the MLA to an age much higher than the normative age of adulthood initiation. An increase in the MLA to 21 years is currently under consideration in a number of regions as a means to reduce tobacco use among adolescents. Although MLA increases have been effective in reducing smoking initiation, prevalence, addiction, and deaths^34^, the results of this study suggest that raising the MLA a few years may not be the ultimate solution to curbing tobacco use. Young adulthood remains a period of exploring one’s identity as an adult, although perhaps to a lesser degree^15^. Thus, raising the MLA for purchasing tobacco products is unlikely to protect young adults who are still exploring their identity by experimenting with adult experiences. In addition, the recent greater increase in smoking initiation among young adults compared to adolescents reduces the potential impact of increasing the MLA by only a few years^35^. A bold step must be taken to raise the MLA to an age where it would be fully effective. The renowned Surgeon General’s Report had already pointed out that 98% of smokers first try smoking by 26 years of age^36^.

This study has several limitations. First, given the cross-sectional nature of this study, causality could not be assessed. Second, social desirability bias may have affected the self-reported measures of cigarette smoking, resulting in the under-reporting of cigarette use. Next, the significant adults considered were limited to parents and teachers, as they were identified as the adults most influential to adolescents; however, other potential role models including adult siblings or relatives have also been reported to influence adolescent behaviors. Furthermore, this study did not consider specifics of the relationships between adolescents and significant adults. For instance, positive relationships with significant adults may lead adolescents to mimic the behaviors of these adults, whereas negative relationships may not.

These findings demonstrate adulthood experiences are associated with adolescent cigarette smoking. The risk of smoking among adolescents with certain adulthood experiences was increased in the presence of significant adults, particularly teachers, who smoked. These results serve as empirical evidence of the rite-of-passage effect of smoking, which may be strengthened by the existing MLA law for accessing tobacco products. The results indicate a bold increase in the MLA and targeted interventions are required for adolescents with both adulthood experience and exposure to adult smoking.

## CONCLUSIONS

Adulthood experiences including tall stature, precocious sexual development, independent living away from parents, and work experience were all associated with cigarette smoking among adolescents. The association between adulthood experience and adolescent cigarette smoking was increased in the presence of significant adults, particularly teachers, who smoked. While some efforts to raise the MLA have been made in recent years, bold increases to raise the MLA by a substantial amount are urgently required.
