# Supplementary material for: Individual and joint association of adulthood experiences and parental or teacher smoking with adolescent cigarette smoking
Source: Tob Induc Dis. 2020 Oct 1;18:83. doi: 10.18332/tid/127519 (PMC7549380; doi:10.18332/tid/127519)
Supplement: Supplementary file 1 [file TID-18-83-s1.pdf]

Table S1 Characteristics of the study participants by smoking status

Characteristics		Total	Non-daily smokers	Daily smokers
		N (%)	N (%)	N (%)
Total		138542 (100.0)	133064 (95.8)	5478 (4.2)
Age (mean ± sd)		15.0 ± 0.01	15.0 ± 0.02	16.4 ± 0.02
Sex (female)		67901 (48.0)	66881 (49.3)	1020 (17.4)
School type				
	Middle	69623 (47.7)	68897 (49.2)	726 (12.6)
	General high	56412 (43.1)	53300 (42.5)	3112 (57.1)
	Vocational high	12104 (9.2)	10496 (8.3)	1608 (30.2)
Household wealth				
	High	47971 (35.0)	46440 (35.3)	1531 (28.3)
	Middle	66515 (47.7)	64110 (47.9)	2405 (43.2)
	Low	24056 (17.3)	22514 (16.8)	1542 (28.5)
Academic performances				
	High	51891 (37.4)	50805 (38.1)	1086 (19.8)
	Middle	38845 (28.1)	37684 (28.4)	1161 (21.4)
	Low	47806 (34.6)	44575 (33.5)	3231 (58.7)
Close friends smoking		59089 (44.3)	53743 (42.0)	5346 (97.5)
Smoking prevention education		82426 (58.7)	79201 (58.8)	3225 (58.0)
Tall stature (≥95th percentile)		7455 (5.6)	7105 (5.5)	350 (6.8)
Precocious sexual development		5852 (4.2)	5464 (4.1)	388 (6.9)
Living away from parents		2725 (1.8)	2520 (1.7)	205 (3.7)
Having a job experience		17945 (13.2)	14642 (11.1)	3303 (59.7)
Parental smoking		64926 (46.2)	61827 (45.8)	3099 (56.2)
Teacher smoking		52854 (37.9)	49368 (36.8)	3486 (64.0)

*Frequency missing: school type (n=403), age (n=526), height (n=3687)
Variables with missing data were treated with the NOMCAR option

Table S2 Association of adulthood experiences and significant adults smoking with daily cigarette smoking

	Daily cigarette smoking		
	Model 1[a]	Model 2[b]	Model 3[c]
	aOR (95% CI)	aOR (95% CI)	aOR (95% CI)
**Adulthood experiences**			
Tall stature	1.26 (1.10, 1.43)	1.27 (1.11, 1.45)	1.23 (1.08, 1.40)
Precocious Sexual development	1.40 (1.21, 1.62)	1.40 (1.21, 1.63)	1.36 (1.17, 1.58)
Living away from parents	1.32 (1.11, 1.57)	1.32 (1.11, 1.58)	1.32 (1.10, 1.57)
Job experience	5.23 (4.88, 5.61)	5.20 (4.84, 5.57)	5.10 (4.76, 5.47)
**Significant adult smoking**			
Parental smoking		1.31 (1.23, 1.40)	
Teacher smoking			1.71 (1.59, 1.83)

a: Model 1 adjusted for age (continuous), sex (male/female), school type (middle/general high/vocational high), close friends smoking (yes/no), participation in smoking prevention education (yes/no), perceived household wealth (high/mid/low), perceived academic performance (high/mid/low)
b: Model 2 additionally adjusted for parental smoking (yes/no) to Model 1
c: Model 3 additionally adjusted for teacher smoking (yes/no) to Model 1

Table S3 Additive interactive effect of adulthood experiences and parental smoking on the risk of adolescent daily cigarette smoking

Adulthood experiences	Daily cigarette smoking				RERI (95% CI); *P*
	Non-smoking parents		Smoking parents		
	N non-daily/ daily	aOR [a] (95% CI);	N non- daily / daily	aOR [a] (95% CI);	
Tall stature [a]					
No	65728/2064	1.00 (Reference)	56828/2780	1.36 (1.28, 1.45)	0.02 (-0.35. 0.40);
Yes	3797/167	1.39 (1.17, 1.66)	3308/183	1.78 (1.50, 2.11)	P=0.906
Sexual development [b]					
Normal	68391/2205	1.00 (Reference)	59209/2885	1.34 (1.26, 1.43)	0.49 (-0.01, 0.99)
Precocious	2846/174	1.36 (1.11, 1.66)	2618/214	2.19 (1.80, 2.68)	P=0.054
Residential status [c]					
With parents	70063/2292	1.00 (Reference)	60481/2981	1.37 (1.28, 1.46)	-0.29 (-0.91, 0.32);
Away from parents	1174/87	1.65 (1.26, 2.16)	1346/118	1.73 (1.36, 2.19)	P=0.358
Job experience [d]					
No	64370/999	1.00 (Reference)	54052/1176	1.34 (1.23, 1.46)	1.16 (0.63, 1.70);
Yes	6867/1380	5.37 (4.88, 5.90)	7775/1923	6.87 (6.25, 7.54)	P<0.001

Covariates adjusted for all models are: age (continuous), sex (male/female), school type (middle/general high/vocational high), close friends smoking (yes/no), participation in smoking prevention education (yes/no), perceived household wealth (high/mid/low), perceived academic performance (high/mid/low)

a: adjusted for sexual development (normal/precocious), residential status (with/away from parents), job experience (yes/no) and the covariates

b: adjusted for tall stature (yes/no), residential status (with/away from parents), job experience (yes/no) and the covariates

c: adjusted for tall stature (yes/no), sexual development (normal/precocious), job experience (yes/no) and the covariates

d: adjusted for tall stature (yes/no), sexual development (normal/precocious), residential status (with/away from parents) and the covariates

Table S4 Additive interactive effects of adulthood experiences and teacher smoking on the risk of adolescent daily cigarette smoking

Adulthood experiences		Daily cigarette smoking				RERI (95% CI); *P*
		Hasn't seen teacher smoking		Seen teacher smoking		
		N non-daily/ daily	aOR [a] (95% CI);	N non- daily / daily	aOR [a] (95% CI);	
Tall stature [a]						
	No	77390/1803	1.00 (Reference)	45166/3041	1.83 (1.70, 1.97)	0.23 (-0.20, 0.66);
	Yes	4200/117	1.31 (1.07, 1.61)	2905/233	2.37 (2.02, 2.79)	P=0.294
Sexual development [b]						
	Normal	80470/1875	1.00 (Reference)	47130/3215	1.84 (1.71, 1.98)	0.15 (-0.41, 0.72);
	Precocious	3226/117	1.58 (1.26, 1.98)	2238/271	2.57 (2.15, 3.08)	P=0.593
Residential status [c]						
	With parents	82158/1926	1.00 (Reference)	48386/3347	1.82 (1.69, 1.96)	0.81 (0.05, 1.58);
	Away from parents	1538/66	1.21 (0.91, 1.63)	982/139	2.85 (2.24, 3.61)	P=0.037
Job experience [d]						
	No	75971/834	1.00 (Reference)	42451/1341	1.78 (1.63, 1.96)	2.74 (2.01, 3.48);
	Yes	7725/1158	5.37 (4.82, 6.00)	6917/2145	8.90 (8.05, 9.84)	P<0.001

Covariates adjusted for all models are: age (continuous), sex (male/female), school type (middle/general high/vocational high), close friends smoking (yes/no), participation in smoking prevention education (yes/no), perceived household wealth (high/mid/low), perceived academic performance (high/mid/low)

a: adjusted for sexual development (normal/precocious), residential status (with/away from parents), job experience (yes/no) and the covariates

b: adjusted for tall stature (yes/no), residential status (with/away from parents), job experience (yes/no) and the covariates

c: adjusted for tall stature (yes/no), sexual development (normal/precocious), job experience (yes/no) and the covariates

d: adjusted for tall stature (yes/no), sexual development (normal/precocious), residential status (with/away from parents) and the covariates

© 2020 Kang H. and Cho S.
